# Supplementary material for: Selective Anti-Cancer Effects of Plasma-Activated Medium and Its High Efficacy with Cisplatin on Hepatocellular Carcinoma with Cancer Stem Cell Characteristics
Source: Int J Mol Sci. 2021 Apr 12;22(8):3956. doi: 10.3390/ijms22083956 (PMC8069277; doi:10.3390/ijms22083956)
Supplement: Supplementary file 1 [file ijms-22-03956-s001.pdf]

Supplementary Materials

Figure S1

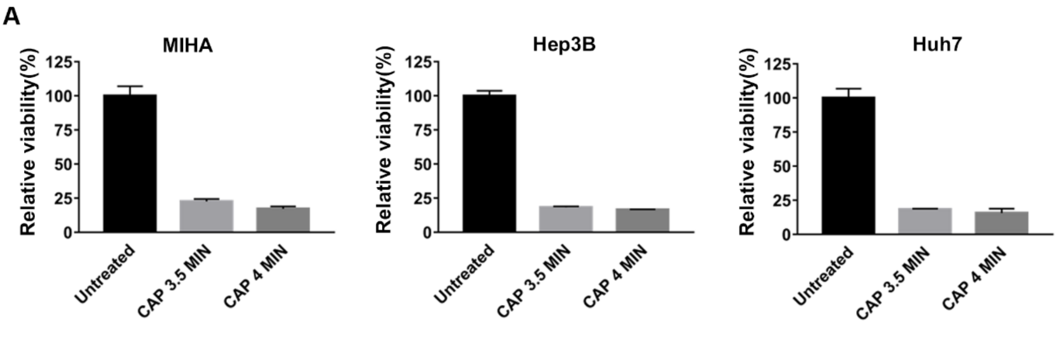

Fig. S1. Increased CAP exposure time in PAM decreased the viability of MIHA as well as Hep3B and Huh7 cells.

(A) 5×10^4 cells of MIHA, Hep3B, Huh7 cells were seeded in 35 mm culture dishes and pre-incubated for 18 h. The cells were treated with PAM exposed with CAP for 3.5 and 4 min and cell viability was analyzed at 72 h using MTT assay. The relative viability was calculated as the ratio of the viability of the treated to the untreated at 72 h. The results are plotted as mean ± SD of three independent experiments.

Figure S2

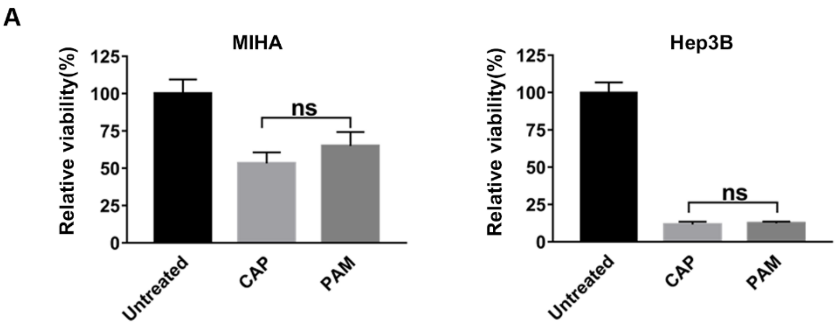

Fig. S2. CAP and PAM showed a highly efficient anti-proliferative effect in Hep3B cells compared to MIHA in response to an increase in cell density.

(A) 10^5 cells of MIHA and Hep3B cells were seeded in 35 mm culture dishes and pre-incubated for 18

h. Cells were exposed to CAP for 2.5 min or treated with CAP-exposed medium, PAM. Cell viability was analyzed at 72 h using MTT assay. The relative viability was calculated as the ratio of the viability of the treated to the untreated at 72 h. The results are plotted as mean ± SD of three independent experiments. * $P < 0.05$ and ** $P < 0.01$ indicate significant difference. ns, not significant.

Figure S3

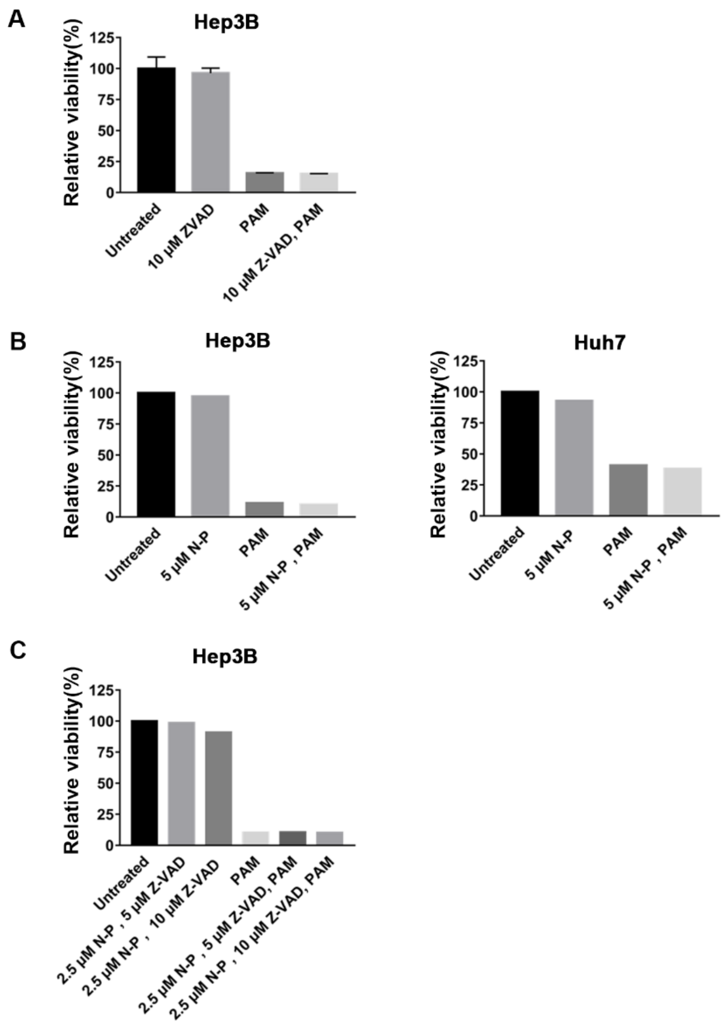

Fig. S3. The inhibitors against caspase and(or) AIF did not recover the decreased viability induced by the PAM treatment in Hep3B

(A-C) Hep3B and Huh7 cells seeded in 35 mm culture dishes were pre-incubated for 18 h. (A) Hep3B cells were pretreated with the pan-caspase inhibitor 10 μM Z-VAD for 1h, followed by the PAM treatment. (B) Hep3B and Huh7 cells were pretreated with the AIF inhibitor 5 μM N-P for 1 h, and then

treated with PAM. (C) Hep3B cells were pretreated with indicated concentrations of N-P and Z-VAD in combination, followed by the PAM treatment. (A, B, C) These treated cells were incubated further for 72 h and the cell viabilities were measured by MTT assays.

Figure S4

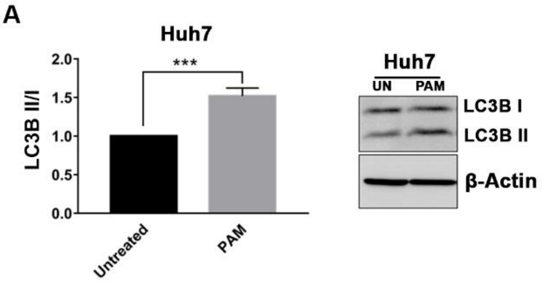

Fig. S4 Autophagic cell death might be induced in PAM treated Huh7 cells.

(A) After PAM treatment (PAM), Huh7 cells were further incubated for 72 h. Untreated (Un) cells were used as a negative control. LC3B processing was monitored by western blot analysis and the expression of LC3B type I and type II is shown (right). The expression ratio of LC3B II/I was normalized using GelQuentNet software (left). β-actin was used as a loading control. The result is presented as the mean ± SD of three independent experiments. * $p < 0.05$, ** $p < 0.01$, and ***$p < 0.001$ indicate significant difference. ns, not significant.

Figure S5

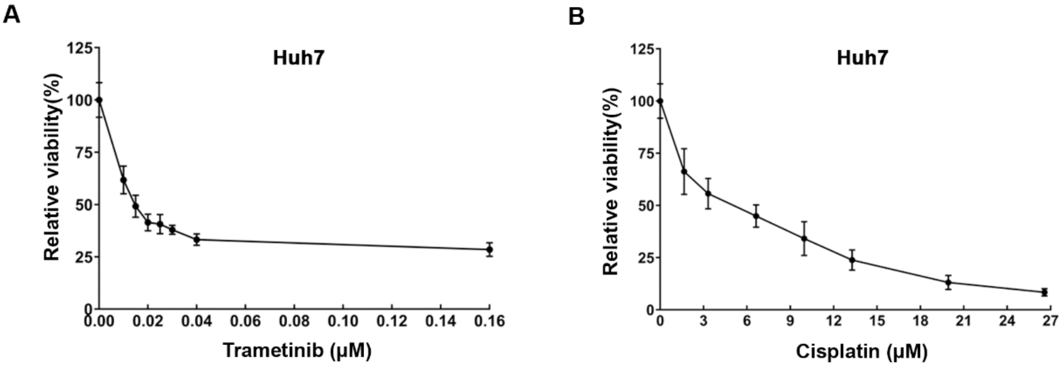

Fig. S5. The IC_{50} of trametinib and cisplatin in Huh7 cells

(A-B) Huh7 cells were seeded in 35 mm dishes, pre-incubated for 18 h, and treated with indicated concentrations of trametinib and cisplatin for 72 h. The viability was analyzed using MTT assay and the relative viability was calculated as the ratio of the viability of the treated to the untreated at 72 h. The relative viability is presented as the mean ± SD of three independent experiments.
